# Assessing the functionality of a water-vending kiosk network with high-frequency instrumentation in Freetown, Sierra Leone^☆^

**DOI:** 10.1016/j.heliyon.2024.e29152

**Published:** 2024-04-08

**Authors:** Matthew S. Falcone, Carlo Salvinelli, Taylor Sharpe, Abrassac Kamara, Evan Thomas

**Affiliations:** a Mortenson Center in Global Engineering and Resilience, University of Colorado Boulder, 4001 Discovery Drive, Boulder, 80301, CO, USA; b Virridy, 1026 Lincoln Pl., Boulder, 80302, CO, USA; c Sierra Leone Millennium Challenge Coordinating Unit, 23 Spur Road, Freetown, Sierra Leone

**Keywords:** Water, sanitation, and hygiene (WASH), Sierra Leone, Water-vending kiosks, WASH intervention functionality, Distributed stored water infrastructure, High-frequency monitoring and instrumentation

## Abstract

Access to safe, reliable, and equitable water services in urban settings of low- and middle-income countries remains a critical challenge toward achieving Sustainable Development Goal 6.1, but progress has either slowed or stagnated in recent years. A pilot water kiosk network funded by the United States Millennium Challenge Corporation was implemented by the Sierra Leone Millennium Challenge Coordinating Unit into the intermittent piped water distribution network of Freetown, Sierra Leone, as a private-public partnership to improve water service provision for households without reliable piped water connections and to reduce non-revenue water. This study employs the use of high-frequency instrumentation to monitor, model, and assess the functionality of this water kiosk network over 2,947 kiosk-days. Functionality was defined via functionality levels on a daily basis through monitored stored water levels and modeled water withdrawals. The functionality levels across the kiosk network were found to be 34% *operational*, 30% *offline*, and 35% *empty*. Statistically significant (p<0.001) determinants of functionality were found for several predictors across the defined thresholds. Finally, modeling of water supply, water demand and withdrawal capacity, and water storage was conducted to further explain findings and provide additionally externally relevant support for kiosk operations.

## Highlights

• Freetown, Sierra Leone lacks continuous access to safe, piped water supply.
• Study uses high-frequency instrumentation to monitor functionality of water kiosk network over 2,947 kiosk-days.
• 34% operational, 30% offline, 35% empty.
• Modeling of water supply, water demand and withdrawal capacity revealed use and service trends.

## 1 Introduction

### 1.1 Background

Water service provision in urban settings of low- and middle-income countries (LMICs) is an increasingly important context toward the achievement of universal access to drinking water, set by Sustainable Development Goal 6.1 [1]. According to the UNICEF-WHO Joint Monitoring Program, only a 1% improvement in access to safely managed drinking water services was observed in urban settings during the first five-year period after the Sustainable Development Goals were announced [2]. In particular, as noted in the 2016 UN Habitat Urbanization and Development Report impoverished countries in Southern Asia and Sub-Saharan Africa have failed to meet the demand of water services as a result of growing urban populations [3]. Furthermore, the IPCC Sixth Assessment Report predicts increasing impacts of climate change on water scarcity, especially for vulnerable groups [4]. The recent trends of slowed or stagnated progress towards water service provision in urban centers of LMICs due to the combination of increasing urbanization and population growth, negative impacts of climate change on water security, and low prioritization and underinvestment in the water sector motivate the need to test and evaluate novel strategies to address urban water services in major cities.

Many cities in LMICs are served by intermittent water systems (IWS), which are piped water networks that are unable to provide reliable and consistent water supply to end users [5]. Intermittency exacerbates microbial contamination [6], forces the use of alternative sources [7] which often exhibit lower water quality and higher cost [8], necessitates household storage [9] which risks recontamination [10], and results in a change of daily routines with negative impacts on household finances, education, health, and livelihoods [11], thus disproportionately impacting the most vulnerable urban households, as is the case in Freetown, Sierra Leone [12].

The Guma Valley Water Company (GVWC) piped water distribution network, commonly referred to as “Guma Water,” is the primary water supply system of Freetown, Sierra Leone, and operates as an IWS. Water mains serving different areas of the Freetown are routinely closed according to a planned water rationing scheme to more equitably provide water across Freetown's geographic areas. However, the Western section of the city where the supply network begins often has better water supply, while Eastern Freetown regularly runs dry [13]. The rationing system is a result of water shortages stemming from the aging and now undersized Guma Dam [14] which at times only meets 54% percent of Freetown's water demand. Further challenges to universal piped water access in Freetown include the limited extent of the bulk water transmission system [15] that does not extend throughout the GVWC service mandate [16], a piped water system that does not reach all service areas and that leaks from network of shallow plastic “spaghetti” pipes, resulting in technical and commercial non-revenue water losses [14], and finally, the inability for many low-income households to afford piped connections. Thus, only about one-quarter of Freetown's residents have access to a piped water connection [14], and inadequate water pressure across many parts of the city further worsens the challenge of water service provision. The GVWC also operates a small fleet of water trucks called “bowsers”, which are intended for emergency refills of critical water storage infrastructure, but are often used within daily operations as a result of the current conditions. The bowsers have also historically been used to provide water supply for households that can afford higher service levels, which may contribute to increased inequity in water services. The poor access to water services experienced by residents of Freetown, especially in low-income areas, motivates the need to develop and test novel solutions that can support basic water service delivery even within this complicated water supply context.

One method of managing water service provision in cities with IWS is the use of water kiosks enabling distributed buffering of stored water. Water kiosks can take many forms, but are typically characterized by two major elements: first, infrastructure for water storage and collection, such as cistern tanks and small superstructures, and secondly, a manual or automated vending system for providing water to users either at low cost or no charge. Water kiosks often operate within formal or informal public-private partnerships, in which public water utilities provide water supply at bulk rates, and private operators sell water to customers on a cost-recovery basis or for minimal profit. Water kiosk networks can benefit communities by providing storage to buffer against water shortages, treating water at the point of collection, and by serving as a point of water service provision that is safer and more reliable than alternative sources. Although water kiosks may improve water service provision, they do not qualify as “safely managed” on the UNICEF-WHO Joint Monitoring Programme Service Ladder for Drinking Water [17] because they are not able to provide water services on the household premises. Additionally, produced water may not be free of microbial or chemical contamination resulting in a Drinking Water Service Ladder classification for water kiosks as high as “basic plus” and as low as “unimproved”. Furthermore, many water kiosk networks fall short on their intended water service provision goals [18], [19], [20], [11], [21], often due to unsustainable service models or unreliable or insufficient water supply. Water kiosks have been studied across many operating contexts, monitoring activities are often limited to project goals and typically do not include meaningful and externally relevant stored water modeling to inform future activities. Thus, limited research has been conducted utilizing high-frequency monitoring at water kiosk sites to model patterns in distributed stored water or to quantify operational insights.

#### 1.1.1 MCC water kiosk pilot intervention

Between 2016 and 2021, a pilot water kiosk network was funded by the Millennium Challenge Corporation (MCC), a United States bilateral foreign aid agency [22], and implemented by the Sierra Leone Millennium Challenge Coordinating Unit (MCCU) as part of the $44.4M Sierra Leone Threshold Program [23], to address non-revenue water and to provide safe and reliable access to water services for households in low-income communities of Freetown without piped water connections [22].

A total of ten (10) water kiosks were constructed during this pilot: five (5) in the Aberdeen community and five (5) in the Kingtom community. Both of these communities are located in northern Freetown, which receives better water supply than eastern Freetown, but still experiences extreme water shortages. Both communities are home to approximately 2,000 households and populations estimated around 15,000 people each. One kiosk in each community was designed with double 10,000 L tanks installed in parallel, while the remaining four kiosks in each community only had a single 10,000 L tank. All kiosk tanks were installed onto elevated platforms, less than 1 m in height, designed to provide additional hydrostatic water pressure from the storage tank to the water spigots, which serve as the point of collection for customers.

Small water pipes supply the water kiosks from protected manifolds at nearby water pipelines into the tops of the tanks, as well as through a bypass around the tanks. Kiosks were designed to operate using Guma Water as a source: either directly from the manifold while the tanks refilled during times of piped water supply, or from the storage tanks to buffer against the water shortages in the piped network during water rationing. Occasional emergency water shortages in the kiosk storage tanks were to be mitigated by the GVWC bowser fleet.

Within the kiosk superstructure, water was to be treated through inline filters and chlorinators, which relied on constant pressure and flow to operate effectively. Finally, water was to be sold by an attendant inside the kiosk superstructure to customers, who would fill water containers at spigots outside the kiosk.

This kiosk network was designed as a private-public partnership, wherein the MCC funded kiosk construction, the GVWC would serve as the public entity responsible for bulk water supply and ongoing kiosk maintenance, and two private operating companies would be responsible for kiosk operations, buying water from the GVWC at bulk rates and selling water to customers at cost recovery or for a small profit.

### 1.2 Scope and purpose of research study

#### 1.2.1 Primary analysis: determinants of functionality

The primary purpose of this study is to define water kiosk functionality using sensor-based stored water data and to explore the factors that would predict higher functionality rates. It is guided by the research question:

*How can a metric for water kiosk functionality be defined using sensor-based stored water level data? Furthermore, what are the determinants of water kiosk functionality, and how can those predictors be used to optimize kiosk network design?*

This research provides a method by which to quantify the reliability of kiosk water services, while informing the design, planning, and replicability of the kiosk network in this context and in others. Additionally, it evaluates the functionality of this pilot water kiosk network, as delivered, to inform future investment decisions by water utilities and funding agencies to improve the impact of water kiosk interventions. Additional themes of kiosk monitoring interests by key stakeholders included water storage, water supply, and water demand.

#### 1.2.2 Secondary analysis: correlation to observed impacts

A secondary purpose of this study is to explore any relationships between levels of kiosk functionality and the levels of household impacts observed in a recent impact evaluation [18] of this pilot water kiosk network. It is guided by the research question:

*Are there any statistically significant and meaningful correlations between observed household impacts of water kiosks and the operational rate and/or distance to nearest water kiosk?*

This secondary analysis seeks to further understand the impacts of the water kiosks on target users, and to elucidate thresholds for kiosk spacing and minimum functionality rate that could possibly impact kiosk network effectiveness at the household level.

## 2 Material and methods

### 2.1 Ultrasonic sensors for water level data

Data for this study were collected using in-situ, high-frequency, remotely-reporting, near-real-time ultrasonic “cistern” sensors developed by (Virridy, Inc.) specifically for this study. These sensors were designed to measure the distance from the water kiosk tank lids down to the stored water level. The sensor platforms transmitted distance readings every 40 minutes, along with the associated time stamp. Data were uploaded via satellite twice per day by a co-located gateway. The sensor platforms incorporated weatherized, tight beam ultrasonic distance sensors (Maxbotix MB7389) with a sensing range between 30 cm and 500 cm. The sensor was used in analog voltage mode, which provides 5 mm resolution within the near range (30-50 cm) and an accuracy of +/- 1% at longer distances, as per stated manufacturer specifications. The sensor was selected for its low cost, low power requirements, weatherization, and tight beam pattern. Over the 5 m sensing range, the sensing beam is constrained to a radius of 30 cm.

A field pilot sensor deployment in Freetown informed a remote codesign between the research team and the MCCU, resulting in improved sensor performance and custom installation hardware. Following manufacture and prior to deployment, each distance sensor platform was tested using a laboratory water column to verify the stability of the output. A simple calibration equation was used to convert voltages to distance readings, and was consistent across all tested sensors in accordance with manufacturer guidelines. During the full deployment, sensors were installed on removable storage tank lids by the MCCU, with the ultrasonic sensor facing directly downward with unobstructed view of the water surface. The installation process required two 34-inch holes to be drilled in the top and bottom of the tank lid, after which the sensor was installed on the surface with an integrated gasket to prevent water intrusion. The sensor communicates wirelessly with a co-located satellite gateway (Virridy, Inc.) which is installed within 30 ft of the sensor with a clear view of the sky. A troubleshooting period, led by the MCCU and the GVWC, took place directly after sensor installation to address physical issues relating to water condensation on sensors, gateway power failures, and connectivity problems. Continued maintenance on sensor systems was conducted by the GVWC, which primarily included swapping or manual resetting of malfunctioning sensors. Installation, troubleshooting, and maintenance was supported by the research team and Virridy.

### 2.2 Data import, cleaning, and processing

**Figure 1:**
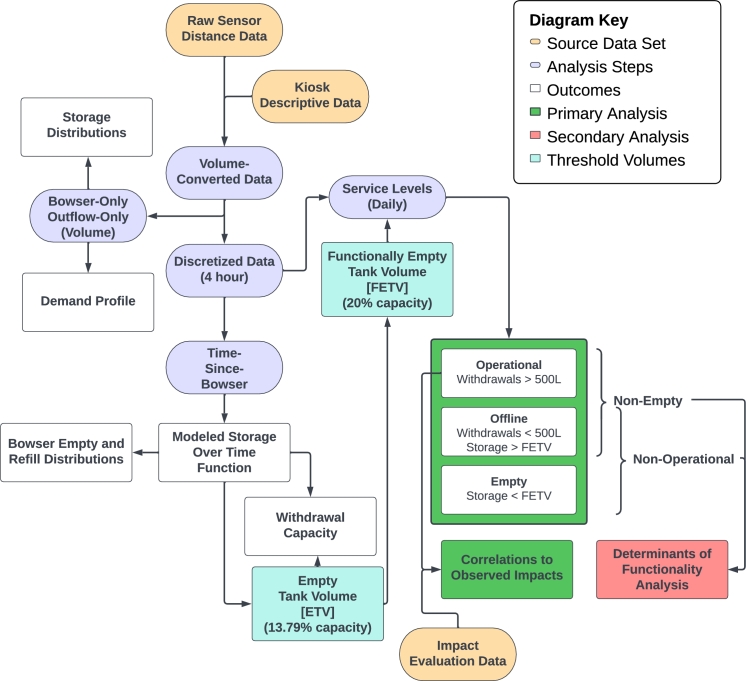
Overview of data analysis including source data, analysis steps, outcomes, primary analysis, and secondary analysis.

All sensor data import, cleaning, processing, and analysis was completed using the R programming language. An overview of the analysis is illustrated in Fig. 1.

Descriptive data about each water kiosk and associated sensors were collected using the mWater data management platform (www.mwater.co). This included kiosk information, including site names, geographic coordinates, and number of 10,000 L storage tanks. It also included sensor details, including identifying information that linked individual sensors to the associated kiosk, installation dates, and dates that sensors were replaced in cases of sensor failure. These data were downloaded from the online platform and incorporated into the raw sensor data. In cases where sensor failures required multiple sensors and/or gateways to be used throughout the period of observation at a single site, the data were stitched together to serve as one cohesive time series in this process.

The start dates at each site were assigned to the earliest confirmed date of kiosk operations and completion of sensor troubleshooting, while the end date was set to the end of the 2022 calendar year for all sites.

Data was inspected and processed for sensor nonfunctionality and dysfunctionality. Sensor nonfunctionality was characterized by unresolvable data drops, during which no data was observed or transmitted due to technical issues that required remote or manual resets, low power, or poor network connectivity. Furthermore, days with insufficient frequency of observations were removed from the analysis to maintain high data quality standards. Sensor dysfunctionality was characterized by observed and transmitted data with unclear signals resulting in uninterpretable temporal trends of water storage levels due to the observed water condensation on the ultrasonic sensors, which are known to cause spurious readings. Data processing was conducted to remove noise from the sensor observations through a distance range filter which retained only values within expected design parameters, and two types of spike filters which either removed extreme values compared to a rolling median or replaced extreme values with the mean of neighboring values. Finally, distance measurements were converted into kiosk tank stored water volumes through a calibration curve based on tank geometry and tank volume, and bounded using the minimum and maximum reliable measurements. This resulted in a processed, volume-converted time series data set, which was used for all further analysis. Since monitoring stored water levels can only observe changes in net outflow and may not observe concurrent inflows and outflows, the analysis in this research was based on differences in net volumetric differences rather than flow rates.

### 2.3 Time series data analysis

Time series data was discretized into 4-hour periods (six periods per day) as a smoothing mechanism to address minor fluctuations in sensor observations. These periods were: late night [00:00 - 04:00), early morning [04:00 - 08:00), late morning [08:00 - 12:00), afternoon [12:00 - 16:00), evening [16:00 - 20:00), and early night [20:00 - 24:00). The mean volume for each time period was used for the primary analysis. This method was chosen over a rolling median, which would have over-smoothed the peaks and valleys within the time series data associated with the sharp emptying and refill patterns, obstructing the underlying trends. It is also more objective than other selective filtration methods and resulted in improved stability of time series data than selective filtration methods. Additionally, it did not smooth measurements across data gaps, providing better time-localized smoothing than alternative methods explored. Although this method aggregates data to lower frequency, it did not negatively impact the primary analysis, which was designed at the daily level. Compared to alternative durations of discretized time periods, the 4-hour periods resulted in time-series data most true to the underlying patterns, while still serving its purpose as a data smoothing method. The indication of activities at the kiosks was determined by differences in volume between consecutive discretized periods.

**Figure 2:**
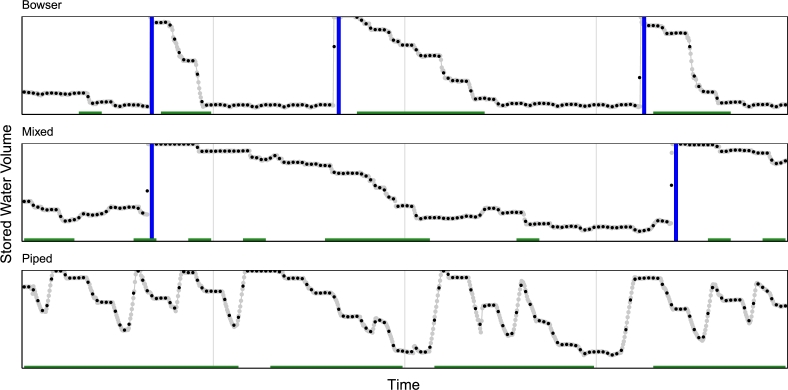
Sample time series data illustrating typical water storage patterns for each water supply scheme over selected 4-week periods. The y-axes demonstrate the entire storage volume for each sample site. Data is provided at the 40-minute frequency (gray) and 4-hour discretized frequency (black), illustrating fidelity of smoothed data to processed data. Vertical blue bars show the nearly-instantaneous bowser refills, and green horizontal bars at the bottom of each time series show days during which operations were identified.

All time series data were inspected and categorized by source water type as shown in Fig. 2: bowsers, which were categorized by infrequent, yet nearly instantaneous refills as observed at sensor reading frequencies; piped, which were categorized by much more frequent, yet much slower refill patterns; and mixed, which included some instantaneous bowser refills but were intermixed with occasional longer piped water refills. Some water kiosk sites only exhibited one water supply scheme throughout the course of this study. However, other sites included multiple water supply schemes at different times of observation. In addition, during this phase of data processing, there was strong indication that bowsers were used as a means of water supply far more than expected by the design of the water kiosk network. It was then confirmed by Freetown partners that bowsers had become the primary water supply mechanism for sites where the piped water network had failed to provide substantial water supply. As a result, data analysis disaggregated for different water supply schemes was incorporated as a major element of this study.

Bowser refills were identified as the last period in a day or within 24-hours in which the net inflow exceeded 2720 L between two consecutive periods. Piped water refill periods were identified as any period in which the net inflow exceeded 400 L. This phase of

For the primary analysis, *operational* kiosks were identified at the daily scale as kiosk-days during which a cumulative net outflow, representing withdrawals, exceeded 500 L across discretized time periods. The daily total withdrawals were estimated as the sum of all consecutive periodized mean volume differences throughout the day. These periods could occur at any time of the day, including before or after bowser or piped refills. The determination of this threshold was related to observed patterns in the sensor data. Volumetric outflows of less than 500 L per day was associated with trends of long, slow leaks without any indication of operations, or with fluctuations in sensor data, while outflows greater than 500 L per day were typically associated with trends of expected daily demand patterns.

In cases of minimal long-term changes in volume with substantial daily fluctuations of similar magnitude from the diurnal evaporation-condensation and temperature cycles, operations were identified by daily withrawals greater than 500 L between mean storage levels of consecutive days.

## 3 Theory and calculation

The times during which kiosks were filled only by bowsers provided an opportunity to analyze the demand-side of kiosk operations and model water withdrawals, because only withdrawals occurred within the long duration of time between nearly instantaneous bowser refills, rather than patterns of superimposed water supply and withdrawals. The bowser-only time series data set was sliced at each identified refill, reparameterized from date-and-time format to time-since-bowser refill, and filtered to remove any sites that did not receive a refill within 30 days, resulting in 110 time segments totalling 1315.8 kiosk-days.

A non-zero Empty Tank Volume (ETV) estimate was developed as the measure of the lowest storage volume allowed by tank geometry that provides enough water pressure to support operations. This level is a result of hydrostatic stored water pressure and friction losses within the system as they relate to customer demand and operations. It includes any dead water below the tank outlet as well as water prevented from draining due to pipe friction in the kiosk. The ETV estimate was quantified through modeling of bowser-site withdrawals over time.

The observed water storage volume with respect to time was modeled by an exponential decay function [Equation: (1a)] fit to the median water storage level at each discretized time-since-bowser refill across all time segments. This function was initialized to fit the median stored water volume (V) at any given time in days since last bowser refill (t) to the difference between the known 10,000 L Full Tank Volume (FTV) and the unknown Empty Tank Volume (ETV), multiplied by the exponent of the product of time (t) and a negative decay constant (*α*), plus the Empty Tank Volume (ETV). The Empty Tank Volume (ETV) was fitted to the asymptote at 1,379 L (p < 2e-16) and the decay constant (*α*) to 0.3897d^−1^ (p < 2e-16). The resulting exponential decay formula [Equation: (1b)] was used to predict the kiosks water storage level based on time since last refill (pseudo-R^2^ = 0.974).

(1a) $$V(t)=(FTV-ETV)e^{-\alpha t}+ETV$$

(1b) $$V(t)=(10,000L-1,379L)⁎e^{\left(-0.3897d^{-1}\times t\right)}+1,379L$$

The observed water withdrawal rate with respect to time since-last-bowser refill [Equation: (2a)] was modeled by the additive inverse of the derivative of the water storage over time function [Equation: (1a)].

(2a) $$V^{\prime }(t)=\alpha (FTV-ETV)e^{-\alpha t}$$

(2b) $$V^{\prime }(t)=0.3897d^{-1}(10,000L-1,379L)e^{-0.3897d^{-1}\times t}$$

The withdrawal capacity with respect to volume function [Equation: (3a)] was derived by rearranging the water storage over time function [Equation: (1a)] in terms of time and substituting it into the additive inverse of its derivative [Equation: (2a)].

(3a) $$V^{\prime }=\alpha (V-ETV)$$

(3b) $$V^{\prime }=0.3897d^{-1}(V-1,379L)$$

The Functionally Empty Tank Volume (FETV) is the lowest storage volume at which kiosks are typically able to maintain sufficient withdrawals to support meaningful demand before ceasing operations, and is the threshold at which kiosks were considered *empty* in the primary analysis. This is more subjective than the ETV, and is more closely related to the withdrawal capacity.

Withdrawal capacity, the physical rate at which water can be drawn from the kiosk, decreases with the hydrostatic water pressure from stored water level, likely generating a feedback loop in which increasing queue lengths and fill times drive customers to seek more accessible alternative sources, thus causing kiosks to cease operations. This likely occurs prior to reaching the ETV because the costs of staffing an underperforming kiosk likely outweigh the profit from sales. The Functionally Empty Tank Volume (FETV) was set to 20% of the kiosk storage capacity, a value slightly higher than the Empty Tank Volume.

**Figure 3:**
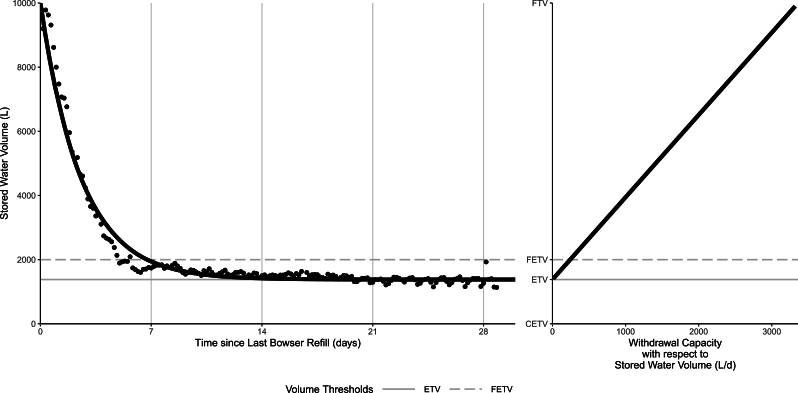
Observed median water storage levels in time since last bowser refill and modeled storage over time function [Equation: (1b)] on left. The withdrawal rate with respect to volume [Equation: (3b)] on right shows a linear increase from the ETV up to its maximum at the full tank, which is extensible to single and double tank sites. Horizontal lines for ETV (modeled asymptote) and FETV (*operational* threshold) estimates are shown in gray.

These modeled storage and withdrawal estimates can be extrapolated across the entire kiosk network when scaled to tank capacity because they describe the relationships between water storage and water demand, and are independent of water supply. The Empty Tank Volume is 1,379 L for single tanks and 2,758 L, and the Functionally Empty Tank Volume is 2,000 L for single tanks and 4,000 L for double tanks. A visualization of the median stored water volumes over time with the fitted model is shown in Fig. 3, with horizontal lines depicting the ETV and FETV, as well as the relationship of withdrawal capacity with respect to stored water volume.

A demand profile estimating the relative demand across hours of the day was developed using the volume-converted data set prior to time-discretization, filtered for only bowser-supply data. Volumetric change was calculated between each set of consecutive data points at each kiosk, then filtered for outflow values only. Since this analysis utilized only outflow data aggregated at the hourly level, sensor measurement fluctuations did not have a major impact on this analysis. Time stamps were rounded down to the nearest hour, and the hours of analysis were truncated to projected hours of kiosk operation, extrapolated from observed withdrawals. The relative proportion of daily demand at each hour was then calculated from the mean value of the volumetric differences at each hour across the entire data set.

### 3.1 Correlation to observed impacts

A recent impact evaluation was conducted to determine the directly attributable impacts of the Freetown pilot water kiosk network on household water security and household water quality [18]. Household surveys were conducted by enumerators trained by and employed by the Sierra Leone Ministry of Water Resources to collect information on household demographics, water source use and preferences, and a measurement of water security using the Household Water Insecurity Experiences (HWISE) scale [24]. Household water quality testing was conducted by water quality technicians trained by and employed by the Sierra Leone Ministry of Water Resources to analyze the temperature, pH, residual chlorine, and microbial water quality through *E. coli* measurements using Aquagenx Compartment Bag Test and associated standard protocols [25] in household stored drinking water containers. Verbal informed consent was obtained from each study participant by enumerators and water quality technicians. The survey and study methodology for the published impact evaluation was reviewed and approved by the University of Colorado Boulder Institutional Review Board (Protocol No. 20-0333) and by the Sierra Leone Research Secretarial Technical Committee on WASH (AP/RC/14072020).

These data were collected at the same households across several sampling rounds: the Baseline observation occurred between August and November 2020, the Midline observation occurred between August and September 2021, and the Endline observation occurred between February and March 2022. The Midline and Endline data were combined into a Combined Endline to account for seasonality. Only households in the Treatment group that self-reported kiosk use in either the Midline or the Endline were included for this correlational analysis between observed water kiosk functionality and associated household-level impacts. Self-reported kiosk use was not directly asked to avoid survey bias; instead water kiosks were included as a water source from which respondents could select as a primary, secondary, or tertiary source of drinking water for the past four weeks. The geospatial coordinates for those households and the entire pilot kiosk network were included in this analysis as well.

Linear regressions were conducted to explore any statistically significant relationships between either outcome variable and any of the predictor variables: household-kiosk distance, the daily frequency rate of kiosk operations, and the distance-operational rate interaction term.

The independent variables in the linear regressions were the changes in HWISE scores and *E. coli* values between the Baseline and the average of the Midline and Endline scores within the same households. They were calculated as the difference between the Baseline value and the mean of the Midline and Endline values for each household. Positive values represent an improvement in conditions, associated with the reduction in surveyed HWISE score toward improved water security and the reduction in measured *E. coli* concentrations toward improved microbial water quality.

The predictor variables included the distance in meters between households and the nearest water kiosk, the *operational* daily frequency rate of the closest water kiosk within the four weeks leading up to the Midline and Endline sampling rounds until the last day of sampling, and the interaction term between those two predictors. The *operational* rate was tested both as a continuous variable and a log-transformed variable.

## 4 Results and discussion

### 4.1 Primary results

#### 4.1.1 Functionality level

**Figure 4:**
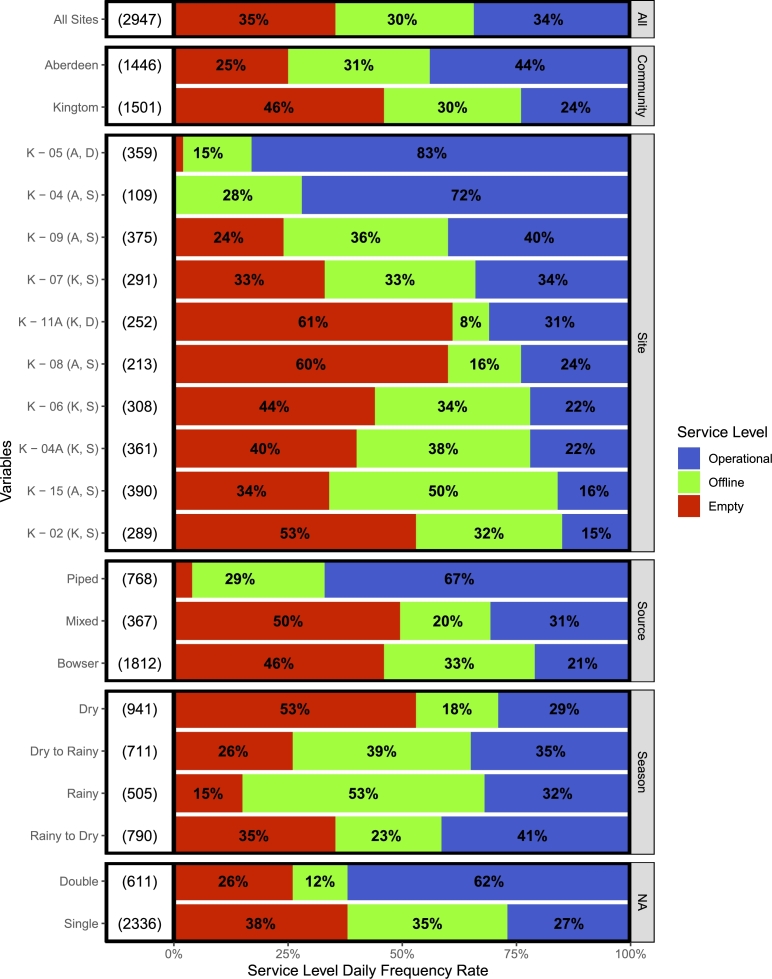
Kiosk functionality levels, desegregated by all sites, communities, each sites, water source, number of tanks, and season with sample size in days (left); all categories except for “Season” ordered by “operational” rate. Kiosk sites labeled by kiosk code, community (A = Aberdeen, K = Kingtom), and number of tanks (S = single, D = Double).

The water kiosk functionality rate is defined by three categorical levels at the daily frequency. The highest functionality level is defined as *operational*, during which observed withdrawals exceeded 500 L throughout the day, enough to meet a meaningful volume of customer demand. The lowest functionality level is defined as *empty*, during which the median periodized water volume at the kiosk was below the FETV threshold of 2000 L for single tanks and 4,000 L for double tanks. The middle functionality level is defined as *offline*, during which less than 500 L of withdrawals per day was observed, yet the median water volume remained above the FETV threshold. The water kiosk functionality level daily frequency rates are illustrated in Fig. 4, disaggregated at meaningful spatial-, design-, and temporal-based parameters, with samples sizes in days provided to the left of the bars. These functionality levels were used to evaluate various parameters as determinants of functionality.

Across all sites, the functionality level rates of the pilot water kiosk intervention were observed in this study to be approximately equal: 34% *operational*, 30% *offline*, and 35% *empty*. Aberdeen was found to exhibit higher *operational* and lower *empty* rates than Kingtom. A wide range of functionality levels were observed between kiosks, with *operational* rates ranging from 15% to 83%, and *empty* rates ranging from 0% to 61%.

The *operational* rates of water kiosks that received piped water supply (67%), on which they were designed, were over three times that of sites that received water supply from bowsers (21%), and unsurprisingly, the *operational* rates of the sites that received mixed water supply fell directly between that of the other two supply schemes (31%). Similarly, the *operational* rate of kiosks constructed with double 10,000 L tanks (62%) were about three times that of single tank sites (21%). The magnitude of these differences provides strong evidence to suggest that piped water supply and 20,000 L storage capacity could result in a highly functional water kiosk network in Freetown.

The differences in functionality level rates across seasons were somewhat more complicated, and are interpreted through a relationship of water supply and demand. *Operational* rates did not change very much across seasons, but *offline* and *empty* rates did. The *empty* rates decreased from the dry season (53%) through the dry-to-rainy transitional season (26%) into the rainy season (15%), and back up during the rainy-to-dry transitional season (35%). Conversely, the *offline* rates increased from the dry season (18%) through the dry-to-rainy transitional season (39%) into the rainy season (53%), and back down during the rainy-to-dry transitional season (23%). The shift between the *empty* and *offline* functionality levels throughout the seasonal cycle is likely due to water supply: when water is more available to supply the kiosks during the rainy season, it is also more available in alternative and often free sources to the customer base, potentially resulting in more kiosk water availability with lower demand.

#### 4.1.2 Determinants of functionality

The determinants of functionality were defined by odds ratios comparing the two thresholds between the three functionality levels. The lower threshold for functionality is *empty* vs *non-empty*, in which the *non-empty* status includes both the *offline* and *operational* functionality levels, providing information about water supply. The higher threshold for functionality is *non-operational* versus *operational*, in which the *non-operational* status includes both the *empty* and *offline* functionality levels, providing information about water services. The lower threshold indicates whether the kiosks can be operated to provide water services, while the higher threshold indicates whether customers can access water from the kiosks.

Odds ratios were calculated independently around both thresholds using four predictors to determine statistical significance and magnitude of the determinants of kiosk functionality. The predictors included the variables: community, which compares the odds ratio of Aberdeen to Kingtom; water source, which compares piped to bowser water supply; season, which compares the rainy to the dry seasons; and the number of tanks, which compares double to single tanks. It should be noted that some of these variables interact; for example, the small number of sites with double tanks were more often supplied by piped water than bowsers. The independent odds ratios and 95% confidence intervals across both thresholds and all predictors are provided in Table: 1 with the associated sample sizes in days and Bonferroni-adjusted (n = 8) Fisher's Exact Test p-values.

**Table 1:** Odds ratios, 95% confidence intervals, and statistical significance of determinants of functionality over both functionality level thresholds. For example, the odds of tanks being *non-empty* are 20.58 times higher for piped-supply kiosks than for bowser-supply kiosks than the odds of tanks being *empty*; p-values given as Bonferroni-adjusted (n=8) Fisher's Exact Test (***p < 0.001, **p < 0.01, *p < 0.05, .p < 0.1).

Variable	n	Empty vs Non-Empty				Nonoperational vs Operational			
		OR	95% CI		p-Value	OR	95% CI		p-Value
Piped vs Bowser	2580	20.58	14.37	30.61	***	7.71	6.39	9.31	***
Double vs Single	2947	1.71	1.40	2.09	***	4.35	3.61	5.25	***
Aberdeen vs Kingtom	2947	2.55	2.18	2.99	***	2.46	2.10	2.88	***
Rainy vs Dry	1446	6.38	4.87	8.46	***	1.17	0.93	1.48	

These results suggest that each of these predictors are meaningful in both magnitude and significance, and should all be considered in updating or expanding the water kiosk network. Most notably and intuitively is the water source: the water kiosk network was designed to operate with water supply from the piped network. Kiosks that did not have piped water supply fared far worse in both water availability and operations than those that did. Thus, ensuring adequate water supply should be the primary focus during future design and implementation of water kiosks in this setting. The second most important factor was water storage capacity: doubling the water storage volume from 10,000 L to 20,000 L allowed for greater buffering capacity against shortages in water supply. Since Guma Water operates as an IWS and bowsers refills are infrequent, additional distributed stored water capacity may allow greater flexibility in water supply for providing reliable water services.

### 4.2 Secondary results

The outcome variables for the logistic regressions included change in HWISE scores and *E. coli* levels within households. The range of observed HWISE scores were 0 to 35 and the range of improvement of HWISE scores within households were -11 to +10. The range of observed *E. coli* levels were 0 MPN/100 mL to 100 MPN/100 mL and the range of improvement in *E. coli* levels within households were -100 MPN/100 mL to + 93.20 MPN/100ML. The minimum household-kiosk distances ranged from 39.44 m to 612.15 m, and the *operational* daily frequency rates for those kiosks during the sampling duration ranged from 11% to 38%.

**Figure 5:**
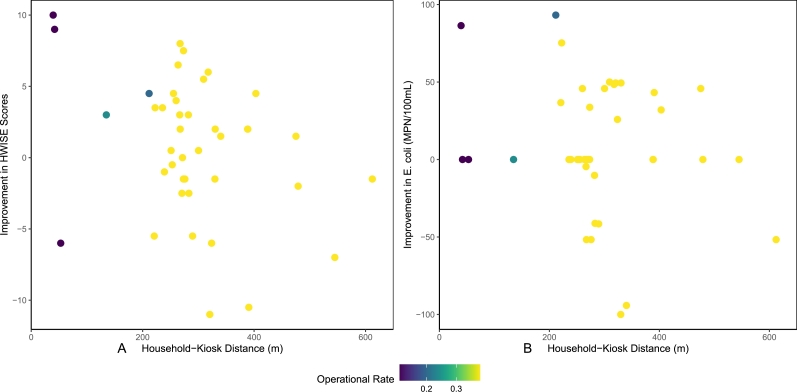
Changes in outcome variables for (A) HWISE; and (B) E. coli over household-kiosk distance, colored by nearest kiosk. *operational* rate.

None of the variables used in the linear regression for correlation to observed impacts analysis were found to be statistically significant predictors (p > 0.15) of the improvement in surveyed HWISE scores or the improvement in measured *E. coli* in household stored drinking water. In absence of statistically significant trends, plots of changes in the outcome variables over household-kiosk distance colored by the *operational* rate of the nearest kiosk have been illustrated in Fig. 5.

The low statistical significance observed in this correlational analysis is likely at least partially due to the low sample size of self-reported kiosk users (n = 41), representing 31.5% of the total 131 Treatment households with complete data sets. It also may be related to the low operational rates observed in this study. If issues observed in the pilot water kiosk network project can be addressed, kiosk operational rates and adoption rates would likely rise, generating a higher likelihood of household impacts and meaningful sample sizes upon which to reconduct this analysis.

### 4.3 Water supply, demand, storage

#### 4.3.1 Water supply

Over the course of this study, only 26% of kiosk-days were supplied by the piped water supply scheme and 12% of kiosk-days were supplied by a mixed supply scheme, while the vast majority, 61% of kiosk-days, were supplied solely by the bowser water supply scheme. This resulted in a gap in the median time between reaching the Functionally Empty Tank Volume (4.25 days) and the median time between bowser refills (10.33 days).

Significantly higher functionality levels were observed at kiosks with reliable piped water supply than at those without piped water access. However, for the vast majority of time, kiosks relied on water trucking, which is neither financially nor operationally feasible for the short- or long-term sustainability in the current context because the kiosk network was not designed to operate on bowser water supply, nor was the bowser fleet prepared to serve as the primary water supply mechanism for the kiosks.

However, the piped water network also lacked the pressure required to supply any water at all to most kiosks. This might be even more problematic in the eastern part of Freetown, where water availability is far worse than in the pilot communities. This issue should be strongly considered in any further replication of this kiosk network in Freetown or in other urban settings that experience pressure drops within their piped water networks. It is recommended kiosks be constructed close to water mains, or that direct pipelines to these kiosks be constructed, according to international standards, as a priority to improve access and equity to the most vulnerable households in these low-income areas to prevent pressure-related water supply failures. If the kiosk network is expanded into areas of Freetown that would be unable to rely on piped water supply, then the procurement and operation of additional dedicated bowsers may be a more effective solution.

#### 4.3.2 Water demand and withdrawal capacity

**Figure 6:**
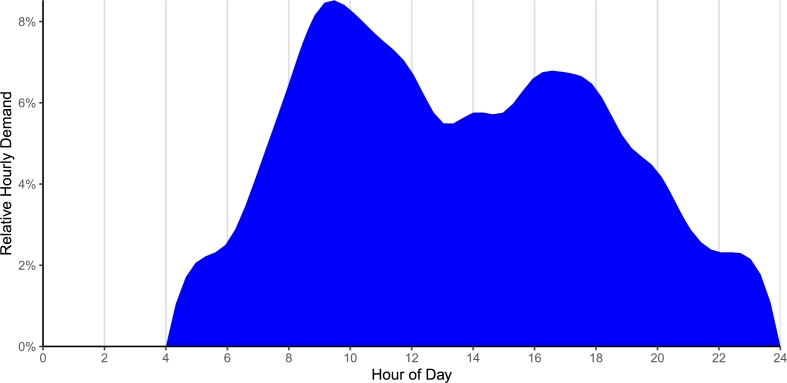
Splined profile of relative demand across hours of the day showing primary peak around 09:00 and secondary peak around 17:00.

The daily demand profile is illustrated in Fig. 6 as a splined area curve of relative rates of demand across hours of operations. Observed demand is minimal during the night, when kiosks are not serviced, and has been truncated from this profile. Met demand begins a meaningful and sharp increase around sunrise at about 06:00 in the morning, which continues until reaching the peak of daily demand around 09:00. It then decreases throughout the late morning and stagnates in the early afternoon, before rising to a smaller secondary peak around 17:00. Afterwards, met demand tapers off until reaching the nighttime baseline.

This daily demand profile can be useful to operators by providing an indication for when additional staffing may be required to optimize operations. Reciprocally, this profile is affected by operations and staffing, without which water withdrawals cannot be made by customers and thus demand cannot be measured. The total water withdrawal at each hour and across the entire day can be estimated by the product of the withdrawal capacity with respect to volume and the demand profile.

The water kiosk withdrawal capacity with respect to stored water volume [Equation: (2b)] demonstrates an order of magnitude linear decrease in withdrawal capacity from 3360Ld at full tanks down to 242Ld at the Functionally Empty Tank Volume, at which point kiosks may cease operations. The maximum observed withdrawal rate likely does not represent actual customer demand in the Aberdeen and Kingtom communities. The installation of additional spigots and larger pipes within the kiosk may help fill customer water storage containers at a faster rate, thus addressing and possibly attracting more customer demand.

Static water pressure from the kiosk water storage tanks was identified as a limiting factor for maintaining flowrate at lower storage levels. One method of addressing this issue would be to maintain higher storage levels in the tanks, but that would reduce the buffering capacity and operational ability of the kiosks. An alternative method would be to increase the static water pressure either by further elevating the storage tanks or by incorporating small booster pumps between the tanks and the kiosk superstructures. The former option could further complicate water supply issues due to low water pressure, but this could also be alleviated by installing smaller ground or subsurface tanks with booster pumps to receive water from the piped network and raise it to the elevated storage tank, which is a standard practice within distributed water storage throughout the world.

#### 4.3.3 Water storage

**Figure 7:**
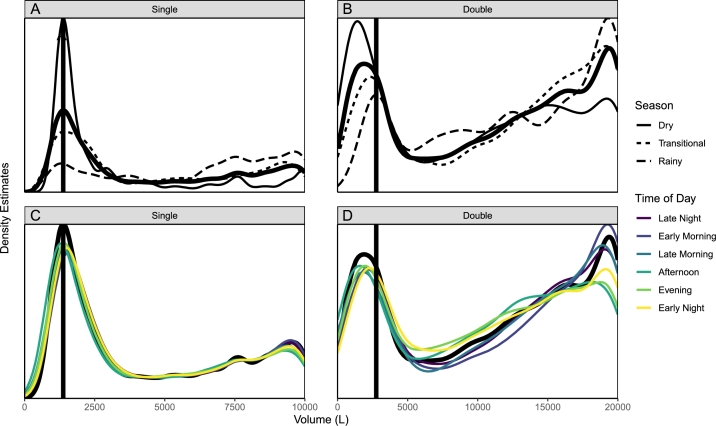
Kiosk water storage distributions with one tank by season (A); two tanks by season (B); one tank by time of day (C); and two tanks by time of day (D). Differences in bimodal distributions demonstrate the positive impact of additional storage on buffering capacity, the rebound potential of kiosk storage during the nighttime, and the impact of the dry season drawing distributions towards empty. Distributions of combined data for each quadrant are illustrated as bold black curves and the Empty Tank Volumes (ETV) are illustrated as vertical black lines.

The distributions of stored water volume across all processed data is shown in Fig. 7 for single and double tanks, and faceted by seasonality and time of day.

Similar non-symmetric bimodal distributions exist across all groups. The first peak, centered near the Functionally Empty Tank Level, is sharp and relatively symmetrical. The second peak is left-skewed towards full: 10,000 L for single-tank sites, and 20,000 L for double-tank sites. The “empty” peak in the single-tank sites is relatively much higher than the “full” peak, while the two peaks are about the same for the double-tank sites. This is likely partially due to the higher proportion of bowser refills at sites with single than double tanks, but also due to improved buffering capacity with double tanks. For both the single- and double-tank sites, the water storage levels during the daytime (06:00 - 18:00] are shifted slightly towards empty and rebound slightly towards full during the nighttime (18:00 - 06:00], further demonstrating buffering capacity. Finally, the distributions tended strongly towards full in the rainy season with the “empty” peak lowering in magnitude and the “full” peak increasing substantially.

Water kiosks with a 20,000 L storage capacity were found to more likely to be *non-empty* and to be *operational* than those constructed with only a 10,000 L capacity. A larger storage volume supports functionality by providing greater buffering capacity against periods of water shortage and by maintaining higher static water pressure for a longer period of time. Given the relatively low cost of acquiring an additional storage tank compared to the construction of an entire kiosk site, it is recommended to design kiosks in this context with two water storage tanks. If source water pressure allows, stacked water tanks would be preferable to further increase hydrostatic water pressure and thus meeting greater customer demand.

### 4.4 Internal relevance

This research provides meaningful insight into the Freetown pilot water kiosk network that can be used by relevant stakeholders to further quantitatively understand kiosk functionality and to inform investment decisions in this context. Overall, the water kiosk intervention was delivered with limited success. The operational rate of the kiosk network was only 34%, and individual kiosk *operational* rates ranged from 15% to 83%. Although the observed functionality of the kiosk network would be unacceptable for a full deployment, the observed partial failures served as useful learning opportunities in a community-scale pilot study from which statistically significant determinants of functionality were developed. Recommendations for improved functionality within this pilot and during full deployment, should Freetown stakeholders deem it a worthwhile endeavor, include components of water supply, water demand, and water storage.

### 4.5 External relevance

Water kiosks can be a useful mechanism for water serice provision in LMICs, especially for servicing low-income communities and/or informal settlements to which governments and water utilities are either unable or unwilling to provide household piped water connections. Water kiosks do not qualify as safely managed drinking water systems on the JMP Drinking Water Service Ladder [17], and thus do not support progress towards the indicator for Sustainable Development Goal 6.1 [1]. However, if properly designed and operated, water kiosks can support progress towards the vision of SDG 6.1 by improving access to and, to some degree, equity toward safe and affordable drinking water. This counterintuitive notion addresses the nuance within this complicated context: although water kiosks are not a solution towards the ultimate goal of improved water service provision, they have the potential to improve access to clean and affordable water in comparison to intermittent water supply and subsequent storage, lower quality water sources, or in areas where technical challenges make the installation and maintenance of on-premises piped water services infeasible.

This research provides externally relevant contributions to the breadth of knowledge about water kiosks by providing relevant frameworks for sensor-based water level monitoring, modeling of distributed stored water, methods to identify kiosk activities and metrics of functionality, and a process by which to assess the relationship of water kiosks to observed household impacts. In particular, the development of a standardized, multilevel metric to assess rates of water kiosk functionality can be used to more fairly assess and compare different water kiosk interventions in the future.

Specific recommendations of this research are highly relevant to the Freetown context, but also provide a starting point for utilities and funding agencies interested in designing water kiosk networks in IWS. It also provides evidence for decision-making around the design and operation of water kiosks, and demonstrates sensor-based techniques to improve water kiosk management to inform investments in the WASH sector. Improved monitoring techniques for water kiosks can be used to optimize design, particularly in environments which lack reliable water supply. The statistical analysis on determinants of functionality can be repeated within future water kiosk evaluations to support design choices across contexts.

### 4.6 Limitations and future work

The success of the Freetown pilot water kiosk network was severely limited by water supply issues. Any future water kiosk iterations or expansions in Freetown should include piped water pressure testing and should consider the installation of direct pipelines from water mains if water is available in sufficient quantities. If not, then alternative mechanisms of water supply, including dedicated rainwater harvesting, increased bowser services, or direct collection and treatment from nearby surface water or groundwater sources should be strongly considered.

The demand profile demonstrated a primary peak of demand in the late morning and a secondary peak in the early evening. However, any potential nighttime demand could not be measured, as it was outside of kiosk operational hours. Future work may include temporary 24-hour per day, 7-day per week staffing of a highly functional water kiosk for an extended period of time in order to develop a 24-hour demand profile. This would inform data-driven decisions around nighttime operations or the adjustment of operational hours to capture more customer demand, thus improving water availability. This analysis could be supported by sales records and operating contracts, which were not available to this research team.

Water treatment mechanisms, including water filtration and chlorination were installed during kiosk construction, but are unlikely to be working optimally due to inconsistent pressure and flow from the storage tanks. An in-depth water treatment process evaluation should be conducted to evaluate finished water quality, assess impacts of hygiene on contamination at the taps, compare raw to finished to raw water quality to quantify improvement, and identify ways to optimize water treatment.

The sensors designed for this study were limited to measuring stored water level, from which the analyses of supply and demand were derived. The integration of in-situ, high-frequency, remotely-reporting, near-real-time inline flowmeters into the kiosk inflow and outflow pipelines with the ultrasonic distance sensors in the kiosk tanks would greatly enhance the ability to conduct quantitative analysis on water kiosk operations. This would improve the identification of kiosk activities, particularly reducing errors in *non-operational* vs *operational*, especially for highly functioning kiosks which often remain full while meeting customer demand. Although bulk flowmeters that monitored water supply from the piped network were installed, apparent measurement errors could not be verified, resulting in the exclusion of this data set from the analysis in this study. In addition, future iterations of ultrasonic sensors should address issues relating to humidity and condensation, increasing the range of environmental operating conditions, thus fully leveraging the 40-minute frequency of data collection. Combined with a 24-hour demand profile, higher accuracy can be used to identify leaks by observing a small but consistent decrease in water level outside of the expected hours of operation. Additionally, a true real-time sensor network can be used beyond evaluation purposes into a more direct operating capability, in which the GVWC or others could monitor water levels across a kiosk network and respond to challenges such as spills or shortages as they occur. Furthermore, the use of these sensors on larger, more centralized storage components, including the set of reservoirs distributed throughout Freetown, could help the GVWC better manage the entire piped water network.

Finally, sensor-based kiosk monitoring alone is unable to ascertain why kiosks were *offline*, a functionality level that occurred 32% of the time. It is possible that kiosks were not staffed, customers chose alternative sources, technical issues or breakdowns occurred, or some other yet unidentified reason. Future qualitative research or mixed-methods study designs can help elucidate a more refined understanding of kiosk operations and kiosk-customer relations to optimize kiosk functionality, and likely impact at the household level.

### 4.7 Conclusions

The Freetown water kiosk pilot served its purpose: to test the design and functionality of a private-public partnership water kiosk intervention in a real-world environment, from which to develop insight and test assumptions. The use of sensor data was useful in understanding quantifiable trends, modeling distributed stored water, and developing statistically significant predictors of kiosk functionality. The application of learnings from this research can and should be applied to future iterations and expansions of water kiosk networks in Freetown and in other contexts.

### 4.8 Ethics statement

The survey and study methodology for the published impact evaluation was reviewed and approved by the University of Colorado Boulder Institutional Review Board (Protocol No. 20-0333) and by the Sierra Leone Research Secretarial Technical Committee on WASH (AP/RC/14072020). The study was provided an Exempt status. Regardless, written Informed Consent was obtained from all participants, all of whom were at least 18 years of age.

## CRediT authorship contribution statement

**Matthew S. Falcone:** Writing – original draft, Visualization, Project administration, Methodology, Investigation, Formal analysis, Data curation, Conceptualization. **Carlo Salvinelli:** Writing – review & editing, Supervision, Project administration, Methodology, Investigation. **Taylor Sharpe:** Writing – review & editing, Resources, Methodology, Data curation. **Abrassac Kamara:** Writing – review & editing, Project administration, Investigation, Conceptualization. **Evan Thomas:** Writing – review & editing, Supervision, Project administration, Methodology, Investigation, Funding acquisition, Conceptualization.

## Declaration of Competing Interest

The authors declare the following financial interests/personal relationships which may be considered as potential competing interests: Evan Thomas reports financial support was provided by 10.13039/100005185Millennium Challenge Corporation via Cooperative Agreement No. 95332419T0005. Evan Thomas reports a relationship with SweetSense Inc. that includes: employment and equity or stocks. If there are other authors, they declare that they have no known competing financial interests or personal relationships that could have appeared to influence the work reported in this paper.

## Data Availability

The data and code used for sensor analysis is posted for public use at https://github.com/SweetSenseInc/FreetownKiosks.
